# Supplementary material for: Effectiveness of Percutaneous Nephrolithotomy, Retrograde Intrarenal Surgery, and Extracorporeal Shock Wave Lithotripsy for Treatment of Renal Stones: A Systematic Review and Meta-Analysis
Source: Medicina (Kaunas). 2020 Dec 30;57(1):26. doi: 10.3390/medicina57010026 (PMC7823824; doi:10.3390/medicina57010026)
Supplement: Supplementary file 1 [file medicina-57-00026-s001.pdf]

Supplement 1. Search terms and results

**EMBASE (1974-171220)**			**MEDLINE (1964-171220)**			**Cochrane library (1945-171220)**		
No.	Search term	No. of article	No.	Search term	No. of article	No.	Search term	No. of article
1	Exp kidney calculi	30351	1	Exp kidney calculi	19876	#1	MeSH descriptor: [kidney calculi] explode all trees	501
2	Exp urolithiasis	57555	2	Exp urolithiasis	40779	#2	MeSH descriptor: [urolithiasis] explode all trees	1021
3	Renal stone.mp.	3038	3	Renal stone.mp.	1932	#3	renal stone	1207
4	1 or 2 or 3	57842	4	1 or 2 or 3	41059	#4	#1 or #2 or #3	1766
5	Shock wave lithotripsy.mp.	6582	5	Shock wave lithotripsy.mp.	5032	#5	shock wave lithotripsy	784
6	SWL.mp.	2163	6	SWL.mp.	1168	#6	SWL	359
7	Extracorporeal shock wave lithotripsy.mp.	5085	7	Extracorporeal shock wave lithotripsy.mp.	3973	#7	extracorporeal shock wave lithotripsy	668
8	ESWL.mp.	4660	8	ESWL.mp.	3100	#8	ESWL	395
9	Exp lithotripsy	11576	9	Exp lithotripsy	11394	#9	MeSH descriptor: [lithotripsy] explode all trees	662
10	5 or 6 or 7 or 8 or 9	17475	10	5 or 6 or 7 or 8 or 9	12549	#10	#5 or #6 or #7 or #8 or #9	1279
11	Percutaneous nephrolithotomy.mp.	7140	11	Percutaneous nephrolithotomy.mp.	2827	#11	percutaneous nephrolithotomy	478
12	PCNL.mp.	3858	12	PCNL.mp.	1398	#12	PCNL	290
13	Exp percutaneous nephrolithotomy	5873	13	Exp percutaneous nephrolithotomy	5114	#13	MeSH descriptor: [nephrostomy, percutaneous] explode all trees	250
14	11 or 12 or 13	7439	14	11 or 12 or 13	5773	#14	#11 or #12 or #13	542
15	Flexible ureteroscopy.mp.	1024	15	Flexible ureteroscopy.mp.	420	#15	flexible ureteroscopy	100
16	Flexible ureterorenoscopy.mp.	473	16	Flexible ureterorenoscopy.mp.	188	#16	flexible ureterorenoscopy	26
17	Retrograde intrarenal surgery.mp.	624	17	Retrograde intrarenal surgery.mp.	234	#17	retrograde intrarenal surgery	58
18	RIRS.mp.	653	18	RIRS.mp.	235	#18	RIRS	49
19	15 or 16 or 17 or 18	2130	19	15 or 16 or 17 or 18	823	#19	#15 or #16 or #17 or #18	167
20	10 and 14	2470	20	10 and 14	1694	#20	#10 and #14	130
21	10 and 19	989	21	10 and 19	366	#21	#10 and #19	62
22	14 and 19	734	22	14 and 19	277	#22	#14 and #19	50
23	20 or 21 or 22	3363	23	20 or 21 or 22	2009	#23	#20 or #21 or #22	194
24	4 and 23	2808	24	4 and 23	1892	#24	#4 and #23	146
25	Stone-free.mp.	5755	25	Stone-free.mp.	3152	#25	stone-free	945
26	24 and 25	1323	26	24 and 25	709	#26	#24 and #25	94
